# Supplementary material for: Comprehensive Annotation and Functional Exploration of MicroRNAs in Lettuce
Source: Front Plant Sci. 2021 Dec 24;12:781836. doi: 10.3389/fpls.2021.781836 (PMC8739914; doi:10.3389/fpls.2021.781836)
Supplement: Supplementary file 2 [file Data_Sheet_8.ZIP › GSM2230749.plot/Lsa-miR398a_Lsat_1_v5_gn_1_100720.1_177_TPlot.pdf]

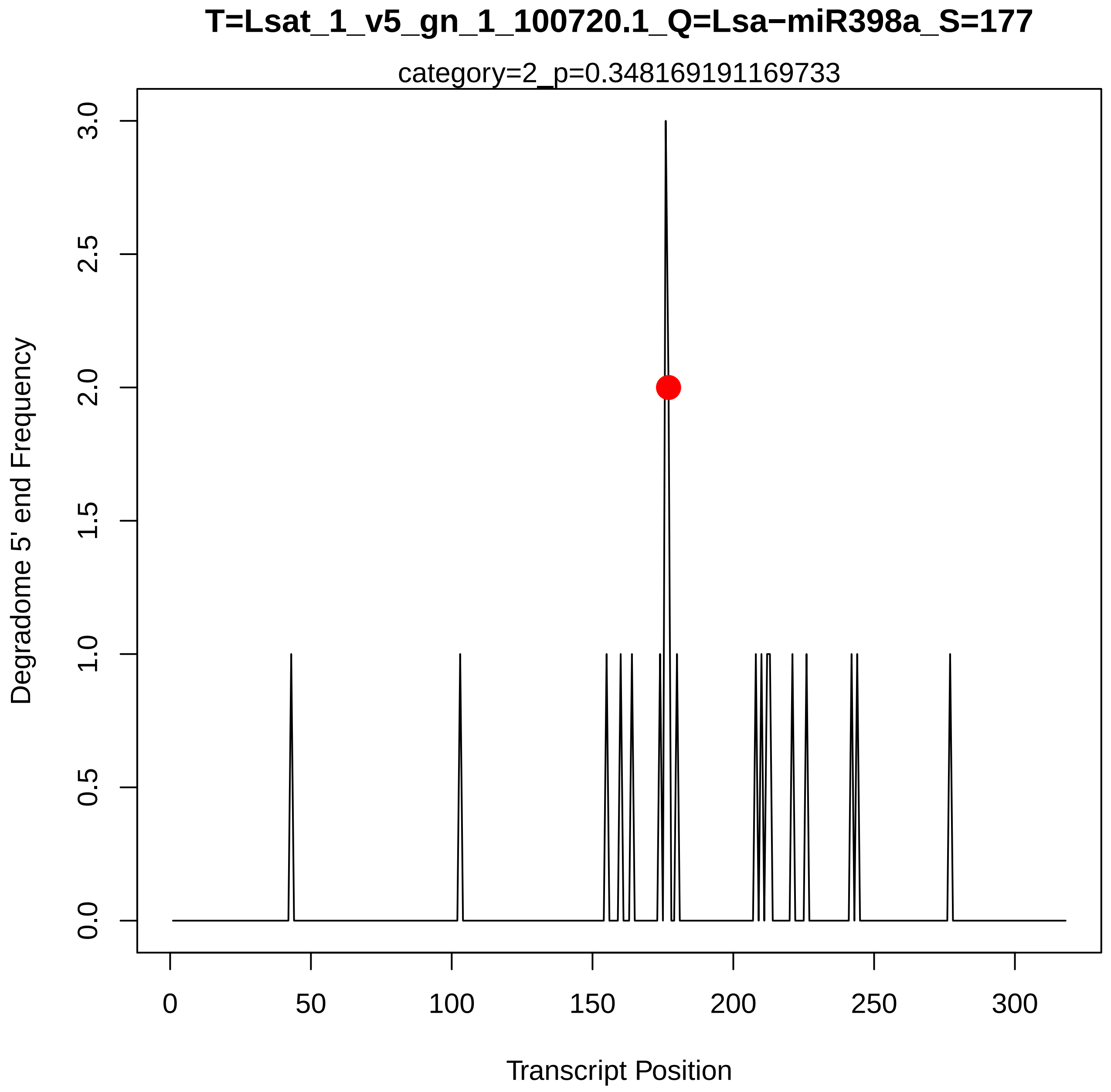

T=Lsat_1_v5_gn_1_100720.1_Q=Lsa−miR398a_S=177
category=2_p=0.348169191169733
Degradome 5' end Frequency
0.0
0.5
1.0
1.5
2.0
2.5
3.0
0
50
100
150
200
250
300
Transcript Position
